# STK39 promotes breast cancer invasion and metastasis by increasing SNAI1 activity upon phosphorylation

**DOI:** 10.7150/thno.62406

**Published:** 2021-06-11

**Authors:** Zhaoping Qiu, Bo Dong, Weijie Guo, Rychahou Piotr, Greg Longmore, Xiuwei Yang, Zhiyong Yu, Jiong Deng, B. Mark Evers, Yadi Wu

**Affiliations:** 1 Department of Pharmacology & Nutritional Sciences, University of Kentucky, College of Medicine, Lexington, KY 40506, United States.; 2 Department of Surgery, University of Kentucky, College of Medicine, Lexington, KY 40506, United States.; 3 Markey Cancer Center, the University of Kentucky, College of Medicine, Lexington, KY 40506, United States.; 4 Department of Medicine (Oncology), Cell Biology and Physiology, Washington University, St. Louis.; 5 Department of Oncology, Shandong Cancer Hospital Affiliated to Shandong University, Shandong Academy of Medical Sciences, Jinan, Shandong, China.; 6 Key Laboratory of Cell Differentiation and Apoptosis of Chinese Minister of Education, Shanghai Jiao Tong University School of Medicine, Shanghai, China.

**Keywords:** STK39, SNAI1, EMT, phosphorylation, stabilization

## Abstract

SNAI1 is widely regarded as a master driver of epithelial-mesenchymal transition (EMT) and associated with breast cancer progression and metastasis. This pro-malignant role is strongly linked to posttranslational modification, especially phosphorylation, which controls its protein levels and subcellular localization. While multiple kinases are implicated in regulation of SNAI1 stability, the precise mechanism by which SNAI1 is stabilized in tumors remains to be fully elucidated.

**Methods**: A series of *in vitro* and* in vivo* experiments were conducted to reveal the regulation of SNAI1 by Serine/Threonine Kinase 39 (STK39) and the role of STK39 in breast cancer metastasis.

**Results:** We identified STK39, a member of Stem 20-like serine/threonine kinase family, as a novel posttranslational regulator that enhances the stability of SNAI1. Inhibition of STK39 via knockdown or use of a specific inhibitor resulted in SNAI1 destabilization. Mechanistically, STK39 interacted with and phosphorylated SNAI1 at T203, which is critical for its nuclear retention. Functionally, STK39 inhibition markedly impaired the EMT phenotype and decreased tumor cell migration, invasion, and metastasis both *in vitro* and *in vivo*. These effects were rescued by ectopic SNAI1 expression. In addition, depletion of STK39 dramatically enhanced sensitivity to chemotherapeutic agents.

**Conclusions:** Our study demonstrated that STK39 is a key mediator of SNAI1 stability and is associated with the pro-metastatic cellular process, highlighting the STK39-SNAI1 signaling axis as promising therapeutic targets for treatments of metastatic breast cancer.

## Introduction

Approximately 90% of cancer deaths are caused by metastasis 1. Metastatic progression spans four distinct steps: invasion, intravasation, extravasation and metastatic colonization 2,3. The development of invasive capability arises from loss of apical-basal polarity and intercellular adhesion in tumor cells. These features are reminiscent of events that occur during EMT, considered a key step during the progression of tumor metastasis 4,5. Extensive studies revealed that the metastasis-linked EMT is controlled by a complex network of transcription factors (TFs), including the SNAI1/SLUG family 6, TWIST 7, δEF1/ZEB1 and SIP1/ZEB2 8,9. Hence, a better understanding of how these TFs regulate tumor metastasis at molecular levels is critical.

SNAI1, a zinc-finger containing transcription factor, induces EMT by direct suppression of E-cadherin (CDH1) transcription during development or tumor progression 6. Studies by us and others demonstrated that SNAI1 expression correlates with tumor grade and predicts a poor patient outcome 10-13. SNAI1 induces resistance to apoptosis, confers tumor recurrence, generates breast cancer stem cell (CSC)-like properties, and induces aerobic glycolysis 14-16. Interestingly, SNAI1 is tightly controlled at both transcriptional and protein levels. Many growth factors and cytokines can transcriptionally regulate SNAI1 expression 17. In addition, SNAI1 is a liable protein, degraded by the ubiquitin-proteasome pathway, despite constitutive mRNA expression. Currently, a number of F-box-containing protein ubiquitin ligases are implicated in the regulation of SNAI1 degradation through kinase-dependent phosphorylation signaling cascades. For example, GSK3β phosphorylates SNAI1 and induces nuclear export, which facilitates β-TRCP-mediated ubiquitination-dependent degradation 12. Meanwhile, other kinases enhance the stability of SNAI1 by inducing nuclear import, nuclear retention or blocking its ubiquitination degradation 18-20. These different types of phosphorylation govern the flexibility and reversibility of SNAI1-mediated EMT.

Despite the number of kinases being linked to SNAI1 stability 18-23, an expanded understanding of the molecular mechanisms that underlie SNAI1 phosphorylation and degradation is needed. In the current study, we assessed the role of STK39 in SNAI1 stability. As a member of STE20-like kinases family, STK39 holds a putative nuclear localization signal and a caspase cleavage site 24. Full-length STK39 exhibits diffuse localization under unstimulated conditions whereas the caspase-cleaved STK39 is located in the nucleus 25. STK39 has been studied for its role in multiple physiological processes, including regulation of chloride and water transport 26, cell transformation and proliferation 25, and cell differentiation 27. Notably, STK39 regulates these physiological processes by phosphorylation-mediated activation 24. In human cancer, STK39 expression is elevated and positively correlated with the adverse tumor stage and poor prognosis in the non-small cell type lung cancer and osteosarcoma 28,29. STK39 is also implicated in regulation of tumor cell proliferation, migration and invasion in multiple cancers, including osteosarcoma and cervical cancer 28,30,31. However, the molecular mechanism that activates the pro-tumorigenic role of STK39 remains largely unknown.

Here, we demonstrate that STK39 interacts with and promotes SNAI1 stability by increasing its nuclear retention. Our results also show that depletion of endogenous STK39 leads to degradation of SNAI1, suppression of EMT and metastasis, which suggests that STK39 is essential for induction of EMT. In addition, depletion of STK39 impacts tumor cell sensitivity to chemotherapeutic agents. Overall, our data uncovers a novel mechanism for a STK39-SNAI1 axis in EMT and further underscores STK39 as a promising therapeutic target for breast cancer treatment.

## Methods

### Plasmids and Reagents

The WT-STK39, KR-STK39 and CA-STK39 were from Jim McCormick (OHSU) and James Wohlschlegel (UCLA). Plasmids of wild-type and deletion mutants for SNAI1 were generated as described 32; all sequences were verified by DNA sequencing. Antibodies purchased from Sigma-Aldrich (St. Louis, MO) include: anti-Flag (F3165), 1:4000; anti-Actin (A2228), 1:10000; anti-Myc (9E10), 1:3000. Anti-STK39 (2281), 1:1000; anti-SNAI1 (3879), 1:1000; and α-Tubulin (2144), 1:1000 were from Cell Signaling (Danvers, MA). N-cadherin (05-915), 1:1000 was from Upstate (Lake Placid, NY). Anti-HA (3F10), 1:10000 was from Roche (Madison, WI) and anti-CDH1 (610181), 1:1000 was from BD Bioscience (San Jose, CA). Anti-Lamin A/C (sc-376248), 1:1000 was from Santa Cruz (Dallas, TX). The p-T203-SNAI1 antibody was from Dr. Greg Longmore. STK39 shRNA expression plasmids were purchased from MISSION shRNA at Sigma-Aldrich. TGFβ1 was from Peprotech. STOCK2S 26016 (STO) was from Tocirs (Minneapolis, MN) and MG132 was from Sigma.

### Cell Culture

The human embryonic kidney HEK293, breast cancer MCF7, MDA-MB-231, MDA-MB-157 cell lines were purchased from the American Type Culture Collection (Manassas, VA) and grown in Dulbecco's modified Eagle's/F12 medium plus 10% fetal bovine serum as described previously 32. The breast cancer cell line T-47D, was grown in RPMI1640 plus 10% FBS. SUM 149 cells were maintained in Ham's F-12 (Invitrogen, Carlsbad, CA) supplemented with 5% FBS, 5 μg/mL insulin, and 1 μg/mL hydrocortisone (Sigma). MCF10A cells were maintained in Dulbecco's modified Eagle's medium-F12 (DMEM/F12) supplemented with 5% horse serum (Invitrogen, 16050122), 1% penicillin/streptomycin (Invitrogen, 15140122), 0.5 μg/ml hydrocortisone (Sigma, H-0888), 100 ng/ml cholera toxin (Sigma, C-8052), 10 μg/ml insulin (Sigma, I-1882), and 20 ng/ml recombinant human EGF (Peprotech, 100-15). All the cells lines were routinely checked for morphological and growth changes to probe for cross-contaminated, or genetically drifted cells. If any of these features occurred, we use the Short Tandem Repeat profiling service by ATCC to re-authenticate the cell lines.

### Invasion and Migration Assay

Invasion and migration assays were performed in Boyden chambers coated with (invasion) or without Matrigel (Migration) as instructed by the manufacturer (BD biosciences). Cancer cell lines were seeded on the top of the upper chamber while the bottom chamber was filled with serum-free culture medium plus 100 nM lysophosphatidic acid. The invasive cancer cells were stained with crystal violent. All experiments were performed in triplicate.

### Immunoprecipitation and Western Blotting

For protein extraction, 5 × 10^5^ cells per well were plated onto six-well plates and transiently transfected with the indicated expression plasmids. At 48 h post-transfection, cells were incubated with or without the proteasome inhibitor MG132 (10 μM) for an additional 6 h before protein extraction and western blot analysis. Primary antibodies against Flag, HA, SNAI1 or STK39 were used for protein detection. For immunoprecipitation, HEK293T cells transfected with the indicated expression plasmids were lysed in buffer (50 mM Tris (pH 7.5; 150 mM NaCl; 5 μg/ml aprotinin; 1 μg/ml pepstatin; 1% Nonidet P-40; 1 mM EDTA and 0.25% deoxycholate). Total cell lysates were incubated overnight with 1 μg of anti-HA or anti-Flag antibody conjugated to agarose beads (Roche) at 4 °C. Lysis buffer washed beads were immunoprecipitated and protein complexes resolved by 10% SDS-PAGE. The western blot was quantified with Image J and normalized with internal loading control (Actin).

### Immunofluorescence Staining

For immunofluorescence microscopy, cells were grown on cover slips, fixed with 4% paraformaldehyde and incubated overnight with anti-Myc, anti-CDH1 or anti-N-cadherin antibody. Proteins were visualized by incubation with goat anti-mouse conjugated with Alexa Fluor 568 (Invitrogen). Finally, cover slips were incubated with 4′, 6′-diamidino-2-phenylindole (Sigma-Aldrich) for 20 min and visualized under a fluorescent microscope.

### Cell proliferation assay

CCK8 proliferation assays (Takara, Japan) were performed to determine the effect of STK39 on proliferation. Transfected cells were seeded in 96-well plates, and cultured at 37 °C in a 5% CO_2_ humidified atmosphere. At selected time points, 10 μL CCK-8 solution was added and cells incubated for 2-4 h at 37 °C. To calculate the number of viable cells, the staining intensity was measured as an absorbance at 450 nm. Results are presented as the means ± standard deviation (SD). Data were based on three independent experiments.

### Quantitative Real-Time PCR

Total RNA was isolated using RNeasy Mini kit (Qiagen, Valencia, CA) according to the manufacturer's instructions. Specific quantitative real-time PCR experiments were performed using SYBR Green Power Master Mix following manufacturer's protocol (Applied Biosystems, Foster City, CA).

### Soft Agar Colony Formation Assay

MDA-MB-231 and MDA-MB-157 cells were pretreated with paclitaxel (PTX), and STO alone or in combination for 48 h. Treated cells were washed and grown in a 24-well plate containing 250 µl of 0.3% agarose in complete medium without drug. Fresh complete medium was replenished every 2 days for a total of 12-15 days. Agarose-embedded cell colonies were stained with 1 mg/ml of Cell Stain Solution overnight. Cell colony formation was quantified using Image J software.

### *In vivo* Tumorigenesis and Metastasis Assay

Female SCID mice (6-8 week old) were purchased from Taconic (Germantown, NY) and maintained under specific pathogen-free conditions. All procedures were approved by the Institutional Animal Care and Use Committee at the University of Kentucky and conform to the legal mandates and federal guidelines for the care and maintenance of laboratory animals. MDA-MB-231-luc cells and corresponding clones with knockdown of STK39 expression were injected via tail vein into 6-week-old female SCID mice. Lung metastasis was monitored by the IVIS bioluminescence imaging system. Data were analyzed using the Student's t-test; a p value <0.05 was considered significant.

### Survival Analyses

For each patient in a data set, a score was calculated as the sum of the products of log2 transformed expression values of *STK39*. Using maximally selected rank statistics (R package maxstat), optimal cut-offs for classification of patients into high-risk or low-risk groups were calculated for each data set. A log-rank test was used to assess the Kaplan Meier survival curves and evaluate statistical significance in OS between risk groups. A p-value of <0.05 was considered statistically significant. The R packages survival and survminer were used for these calculations and for data visualization. All statistical tests were performed using R 3.4.2.

### Statistical Analyses

Differences between two independent groups were calculated using Student's t-test, or one-way ANOVA and Tukey test for multiple comparisons as indicated in the figure legends. P values less than 0.05 were considered statistically significant and are denoted as follows: *<0.05, **<0.01, and ***<0.001. All data were analyzed with GraphPad Prism 5 software.

## Results

### STK39 stabilizes SNAI1

**Figure 1:**
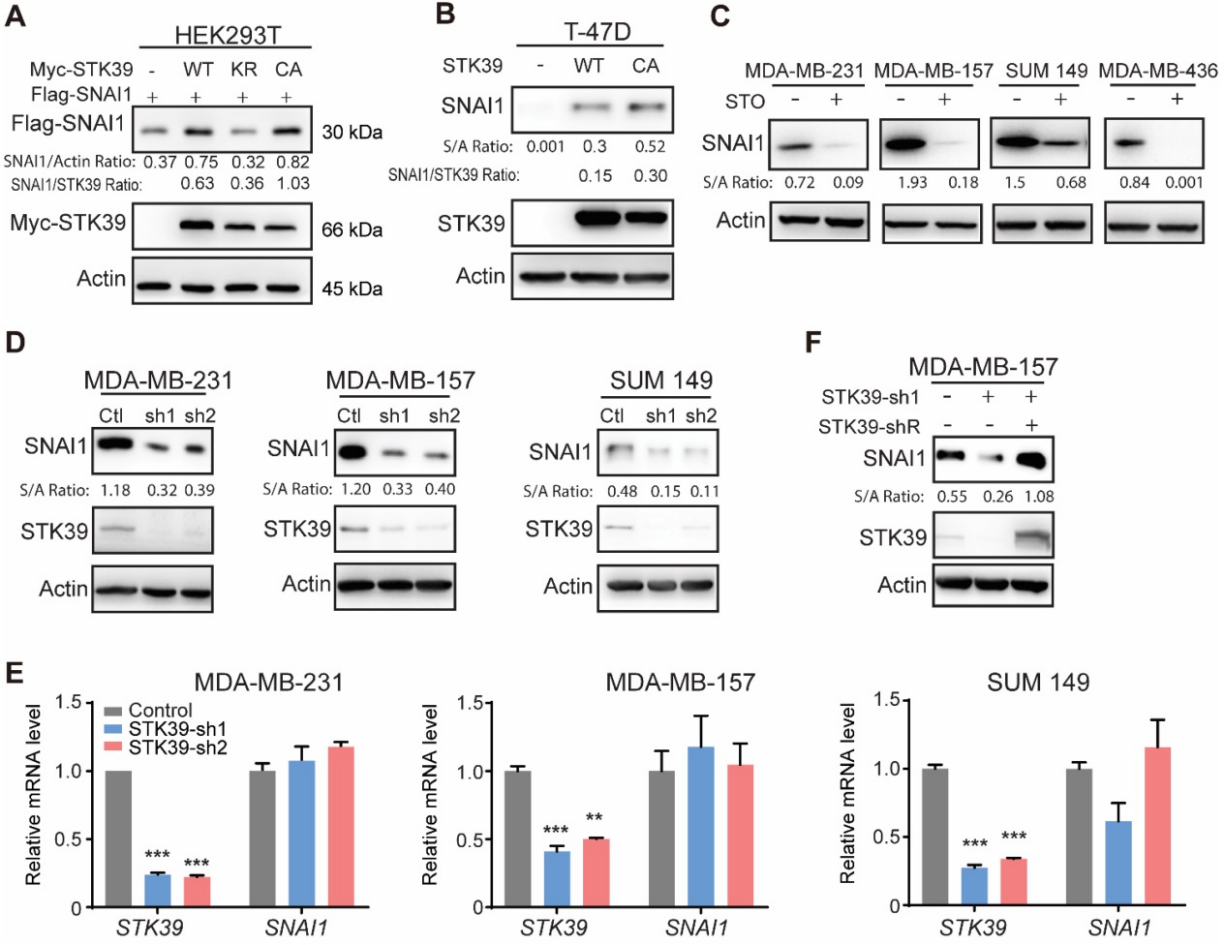
** STK39 stabilizes SNAI1. (A)** Flag-SNAI1 was co-expressed with Myc-tagged STK39 (either wild-type (WT), catalytically inactive KR (K104R), or constitutively active (CA) mutant) in HEK293T cells. Expression of SNAI1 and STK39 were assessed by western blot with Flag and Myc antibodies, respectively. **(B)** WT-STK39 or CA-STK39 was stably transfected into T-47D cells. Lysates were analyzed by western blot. **(C)** Cells were treated with 10 µM STOCK2S 26016 (STO) for 24 h. Lysates were analyzed by western blot. **(D)** The protein expression of STK39 and SNAI1 from MDA-MB-231, MDA-MB-157, and SUM 149 cells transfected with control or two individual STK39 shRNAs was analyzed by western blot. **(E)** MDA-MB-231, MDA-MB-157 and SUM149 cells were transfected with control or two individual STK39 shRNAs. The mRNA was detected by real-time PCR. ** P<0.01, *** P<0.001, compared with controls. **(F)** The protein expression of STK39 and SNAI1 from MDA-MB-157 cells stably transfected with control, STK39 shRNA or STK39 shRNA with shRNA-resistant STK39 (STK39-shR) were analyzed by western blot.

As previously reported 33, by purifying SNAI1 complexes from nuclear extracts of 20 liters HeLa S3 cells expressing Flag-SNAI1 and subsequent top-down mass spectrometry analysis 32, we noticed that STK39 was one of the proteins involved in this complex (data not shown). To investigate the relationship of these two proteins, we co-expressed SNAI1 with STK39 in HEK293T cells. Expression of wild-type (WT) STK39 stabilized SNAI1. A catalytically inactive STK39 harboring the K104R mutation (KR), which functions as a dominant-negative inhibitor of endogenous STK39 34,35, showed no such effect. However, the constitutively active mutant STK39 kinase (T243E/S383D, CA) dramatically increased SNAI1 expression, which indicates that the enzymatic activity of STK39 is required for SNAI1 stabilization (**Figure 1A**). Both STK39 WT and CA also increased endogenous SNAI1 protein levels in T-47D cells, which contain little endogenous SNAI1 (**Figure 1B**). We then treated several breast cancer cells with the STK39 inhibitor STOCK2S 26016 (STO). STO treatment dramatically reduced the SNAI1 expression (**Figure 1C**). Consistent with this, knockdown of endogenous STK39 resulted in a rapid loss of endogenous SNAI1 protein, but had no effect on mRNA levels, in MDA-MB-231, MDA-MB-157 and SUM 149 cells (**Figures 1D and 1E**). To rule out the off-target effect of shRNA, we rescued STK39 expression with shRNA-resistant STK39 in shRNA-mediated knockdown MDA-MB-157 cells. As we expected, ectopic expression of STK39 restored the SNAI1 expression (**Figure 1F**). Together, our results indicate that STK39 stabilizes SNAI1.

### STK39 enhances SNAI1 protein stability by blocking SNAI1 degradation

**Figure 2:**
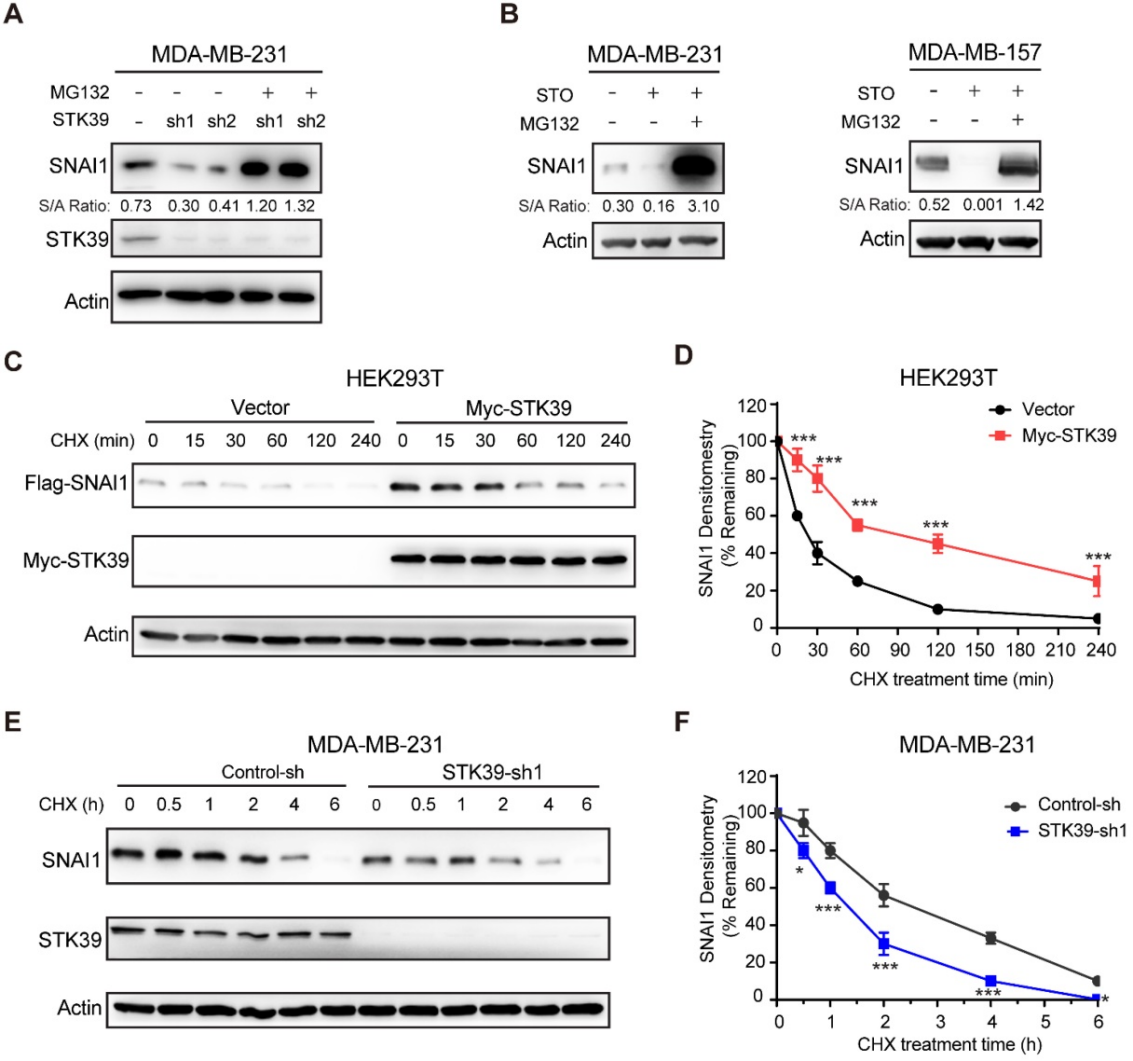
** STK39 blocks SNAI1 degradation. (A)** Protein expression of STK39 and SNAI1 from MDA-MB-231 cells stably transfected with control or two individual STK39 shRNAs and treated with or without 10 μM MG132 for 8 h was analyzed by western blot. **(B)** Cells were pre-treated with 10 µM STO for 1 h then treated with or without 10 μM MG132 for 8 h. Lysates were analyzed by western blot. **(C)** Flag-SNAI1 was co-expressed with vector or Myc-STK39 in HEK293T cells. After treatment with cycloheximide (CHX) for the indicated time intervals, expression of SNAI1 and STK39 was analyzed by western blot using Flag and Myc antibodies, respectively. Presented data are representative of 3 separate experiments. **(D)** The intensity of SNAI1 expression for each time point in (C) was quantified by densitometry and plotted. **(E)** MDA-MB-231 cells were transfected with control or STK39 shRNA. After treatment with CHX as indicated above, expression of endogenous SNAI1 and STK39 was analyzed by western blot. Presented data are representative of 3 separate experiments. **(F)** The intensity of SNAI1 expression for each time point in (E) was quantified by densitometry and plotted. * P<0.05, ***P<0.001, compared with controls.

Because SNAI1 is a liable protein and readily degraded by proteasome degradation and because STK39 stabilizes SNAI1 but has no effect on mRNA expression, we asked whether STK39 blocked SNAI1 degradation. First, we treated STK39 knockdown cells with proteasome inhibitor MG132 and found that the downregulation of SNAI1 in STK39-knockdown MDA-MB-231 cells was restored by MG132 treatment (**Figure 2A**), which indicates that STK39-knockdown facilitates the degradation of SNAI1. Consistent with this, MG132 treatment also restored the SNAI1 expression in STO-treated MDA-MB-231 and MDA-MB-157 cells (**Figure 2B**). We then co-expressed SNAI1 with STK39 or vector control in HEK293T cells and examined SNAI1 degradation. After treatment with cycloheximide (CHX) to block new protein synthesis, SNAI1 degraded rapidly in cells transfected with a control vector (**Figures 2C-2D**). However, SNAI1 levels were stabilized in the presence of STK39 and this effect continued for up 4 h in the presence of CHX. To test whether endogenous SNAI1 is also subjected to similar regulation by STK39, we knocked down endogenous STK39 in MDA-MB-231 cells, and found that endogenous SNAI1 became unstable and degraded rapidly (**Figures 2E-2F**). Since p38, which is activated by STK39 25, was shown to control SNAI1 expression 18, we examined whether STK39 stabilizes SNAI1 through downstream effects in the p38 pathway. Inhibition of p38 by SB203580 resulted in down-regulation of SNAI1 expression 18. However, a similar treatment had no effect on SNAI1 expression enhanced by STK39, indicating that the increase induced by STK39 is p38 independent (**Figure S1A**). To further test whether STK39 blocks the interaction between SNAI1 and GSK3β, we performed an immunoprecipitation with or without STK39. SNAI1 and GSK3β associated to a similar extent in the presence or absence of STK39 (**Figure S1B**). Taken together, these results suggest STK39 leads to SNAI1 stabilization by blocking its degradation.

### STK39 interacts with SNAI1 and phosphorylates SNAI1 on T203

**Figure 3:**
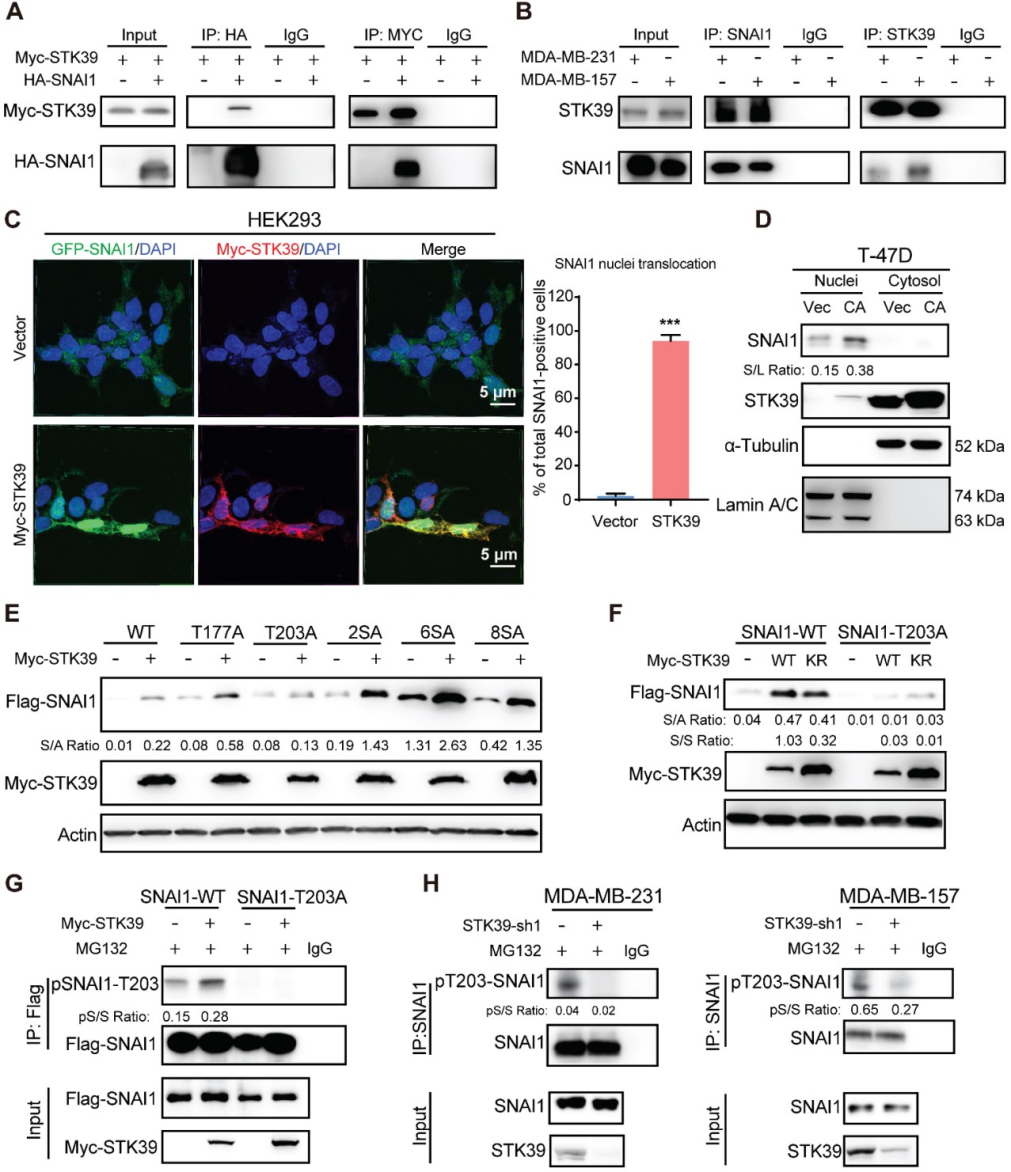
** STK39 interacts with and phosphorylates SNAI1 at T203. (A)** HA-SNAI1 was co-expressed with vector or Myc-STK39 in HEK293T cells. SNAI1 and STK39 were immunoprecipitated (IP) with HA or Myc antibody, respectively, and the associated STK39 and SNAI1 were analyzed by western blot using either Myc or HA antibody. One-fortieth of the lysate from each sample was subjected to western blot to examine the expression of SNAI1 and STK39 (input lysate). **(B)** Endogenous SNAI1 and STK39 were captured by IP from MDA-MB-231 and MDA-MB-157 cells, and bound endogenous STK39 and SNAI1 were examined by western blot. **(C)** GFP-SNAI1 was co-expressed with Myc-STK39 in HEK293 cells. After fixation, the cellular location of SNAI1 (green) and STK39 (red) was examined by immunofluorescent staining using anti-Myc antibody and visualized by fluorescence microscopy (nuclei were stained with DAPI; blue) (left panel). Nuclear SNAI1 was quantified (right panel). *** P<0.001, compared with vector. **(D)** Cell lysates prepared from T-47D cells were transfected with vector or constitutively active (CA)-STK39 and fractioned to identify the location of SNAI1 and STK39. **(E)** Myc-STK39 was co-expressed with Flag-tagged wild-type or different mutants of SNAI1 in HEK293T cells. Protein expressions of STK39 and SNAI1 were analyzed by western blot. **(F)** Myc-STK39 WT or KR was co-expressed with Flag-tagged wild type (WT) or T203A of SNAI1 in HEK293T cells. Protein expressions of STK39 and SNAI1 were analyzed by western blot. **(G)** Flag-SNAI1 WT or Flag-SNAI1 T203A was co-transfected with or without Myc-STK39 into HEK293T cells, then treated with MG132 for 6 h. Cell lysates were immunoprecipitated using anti-Flag antibody and analyzed by immunoblotting using a specific antibody against pT203-SNAI1. **(H)** MDA-MB-231 and MDA-MB-157 cells were transfected with control or STK39 shRNA. Cells were treated with MG132 for 6 h. Cell lysates were immunoprecipitated using anti-SNAI1 antibody and then analyzed by immunoblotting using a specific antibody against pT203-SNAI1.

To delineate the interaction of STK39 with SNAI1, we co-expressed Myc-STK39 and HA-SNAI1 in HEK293T cells and performed a co-immunoprecipitation (IP) experiment. After IP of SNAI1, we detected an associated STK39, and vice versa (**Figure 3A**). IP of endogenous SNAI1 and STK39 from MDA-MB-231 and MDA-MB-157 cells also demonstrated the presence of endogenous STK39 and SNAI1, respectively (**Figure 3B**). To identify the region in SNAI1 that associates with STK39, we generated two deletion mutants of SNAI1 33,36: the N-terminal SNAI1 (amino acids 1-153), which includes the SNAG domain of SNAI1; and the C-terminal SNAI1 (amino acids 153-265), which contains the conserved zinc finger motif (**Figure S2A**). The C-terminal region of SNAI1 was responsible for its interaction with STK39. We then co-expressed Myc-STK39 and GFP-SNAI1 in HEK293 cells. Surprisingly, STK39 stabilized SNAI1 in the nucleus (**Figure 3C**). Although STK39 predominantly localizes to cytosol, it contains a putative nuclear localization signal that enables nuclear translocation 25. Since SNAI1 turnover is decreased in the nucleus, we asked whether stabilization of SNAI1 by STK39 might occur in nucleus and prevent its nuclear-cytosolic transport thereby leading to its stabilization. To test this possibility, we overexpressed STK39 in T-47D cells and fractionated the cells into cytosolic and nuclear fraction. SNAI1 protein was only detected in the nucleus and increased SNAI1 protein was predominantly nuclear in T-47D cells (**Figure 3D**). We then screened potential serine/threonine phosphorylation sites that may facilitate nuclear SNAI1 retention. We co-expressed the WT and mutant SNAI1 with Myc-STK39 in HEK293T cells. STK39 greatly enhanced the stabilization of WT, SNAI1-T177A, SNAI1-2SA, SNAI1-4SA, SNAI1-6SA 12, and SNAI1-8SA (SNAI1-6SA+S105A+S121A), which were resistant to degradation 12 but not T203A 19, which suggests that T203 is a potential target site for STK39 (**Figure 3E**). Consistent with this, STK39 markedly increased the WT-SNAI1 protein levels but expression in SNAI1-T203A did not change significantly (**Figure 3F**). Moreover, using a specific antibody against phosphor-SNAI1 T203 (pT203-SNAI1) 19, we detected SNAI1 phosphorylation in HEK293T cells transfected with wild-type SNAI1, but not SNAI1 T203A (**Figure 3G**, lane 1 vs lane 3). In addition, the pT203-SNAI1 level was upregulated by STK39-WT but not STK39-KR (**Figures 3G** and S2B). Furthermore, endogenous pT203-SNAI1 was detected in MDA-MB-231 and MDA-MB-157 cells, whereas knockdown of STK39 dramatically decreased endogenous pT203-SNAI1 levels (**Figure 3H**). These results consistently indicate that STK39-mediated T203 phosphorylation results in SNAI1 stabilization by nucleus retention thus suppressing SNAI1 degradation.

### STK39 enhances EMT in a SNAI1-dependent manner

**Figure 4:**
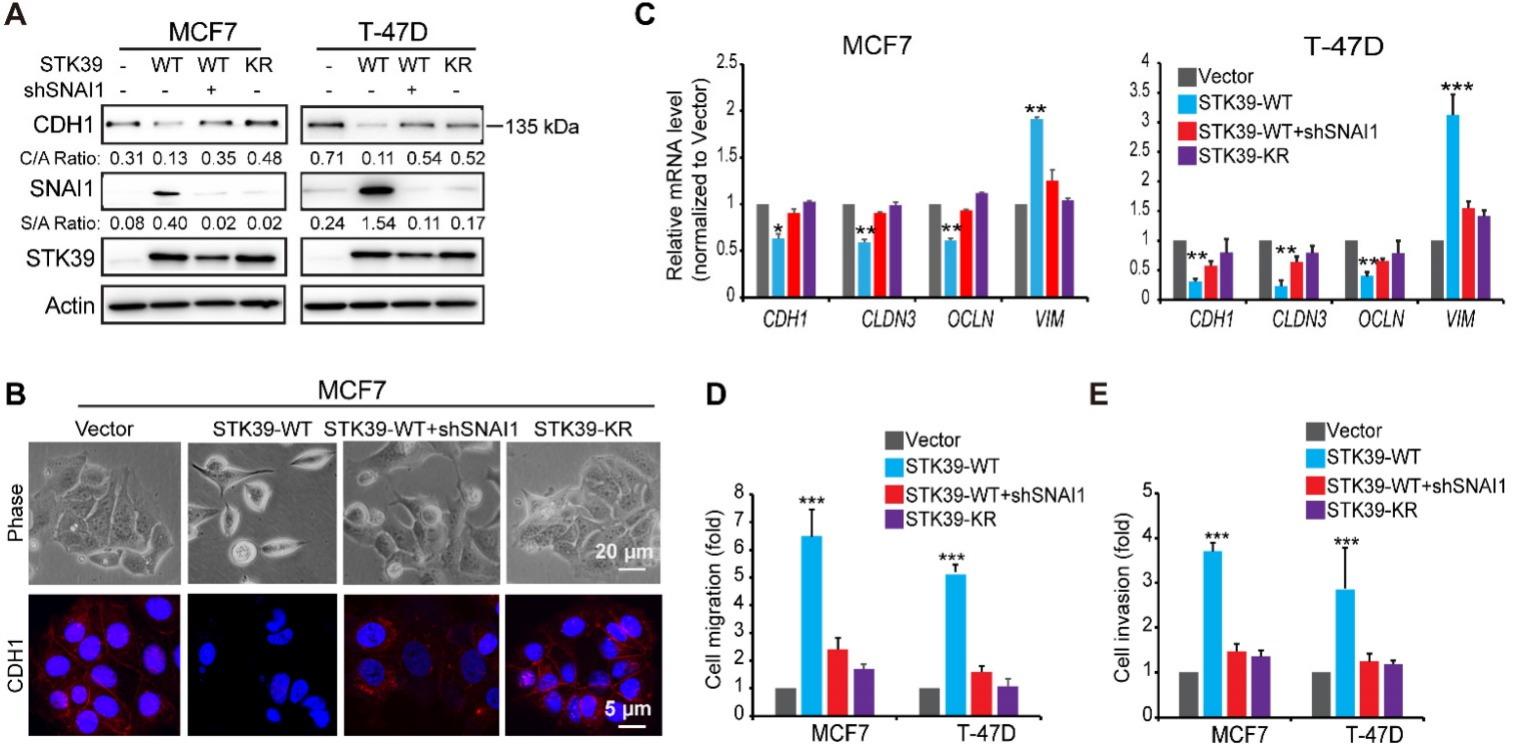
** Overexpression of STK39 induces EMT. (A)** STK39 was expressed in MCF7 and T-47D cells. A rescue experiment with knockdown of SNAI1 expression in WT-STK39 expressing cells was also performed. The levels of CDH1, SNAI1 and STK39 were analyzed by western blot. **(B)** STK39 was expressed in MCF7 cells. A rescue experiment with knockdown of SNAI1 expression was also performed. Morphologic changes indicative of EMT were shown in the phase-contrast images; expression of CDH1 was assessed by immunofluorescent staining. Nuclei were visualized with DAPI (blue). **(C)** STK39 was expressed in MCF7 and T-47D cells. A rescue experiment with knockdown of SNAI11 expression was also performed. mRNA levels were quantitated by real-time PCR. Data are the mean±s.d. of two separate experiments in triplicates. **(D)** Boyden chamber migration assay of modified MCF7 and T-47D cells, as described in A. Data are the mean±s.e.m. **(E)** Boyden chamber invasion assay of modified MCF7 and T-47D cells, as described in A. Data are the mean±s.e.m. * P<0.05, ***P<0.01, *** P<0.001, compared with vector.

To explore the functional role of STK39, we expressed STK39 in two luminal breast cancer cell lines, MCF7 and T-47D. STK39 expression induced SNAI1 stabilization as well as downregulation of CDH1 in these cells (**Figure 4A**). Consistently, STK39 expression induced a morphologic change indicative of EMT (**Figure 4B**), accompanied with downregulation of CDH1. In addition, Real-time PCR revealed that STK39 expression downregulated epithelial markers (CDH1, CLDN3 and OCLN) and upregulated mesenchymal molecules Vimentin (VIM) (**Figure 4C**). Functionally, STK39 expression markedly enhanced the cell migration and invasive capacity (**Figures 4D-4E, and S3**). The catalytic activity of STK39 is required for these functions, because STK39-KR had no effect on SNAI1 expression, the morphological changes, or cell migration and invasion in these cells (**Figure 4 and S3**). Importantly, knockdown of SNAI1 markedly attenuated these changes (**Figures 4 and S3**), indicating that the functional activities promoted by STK39 required SNAI1 upregulation.

**Figure 5:**
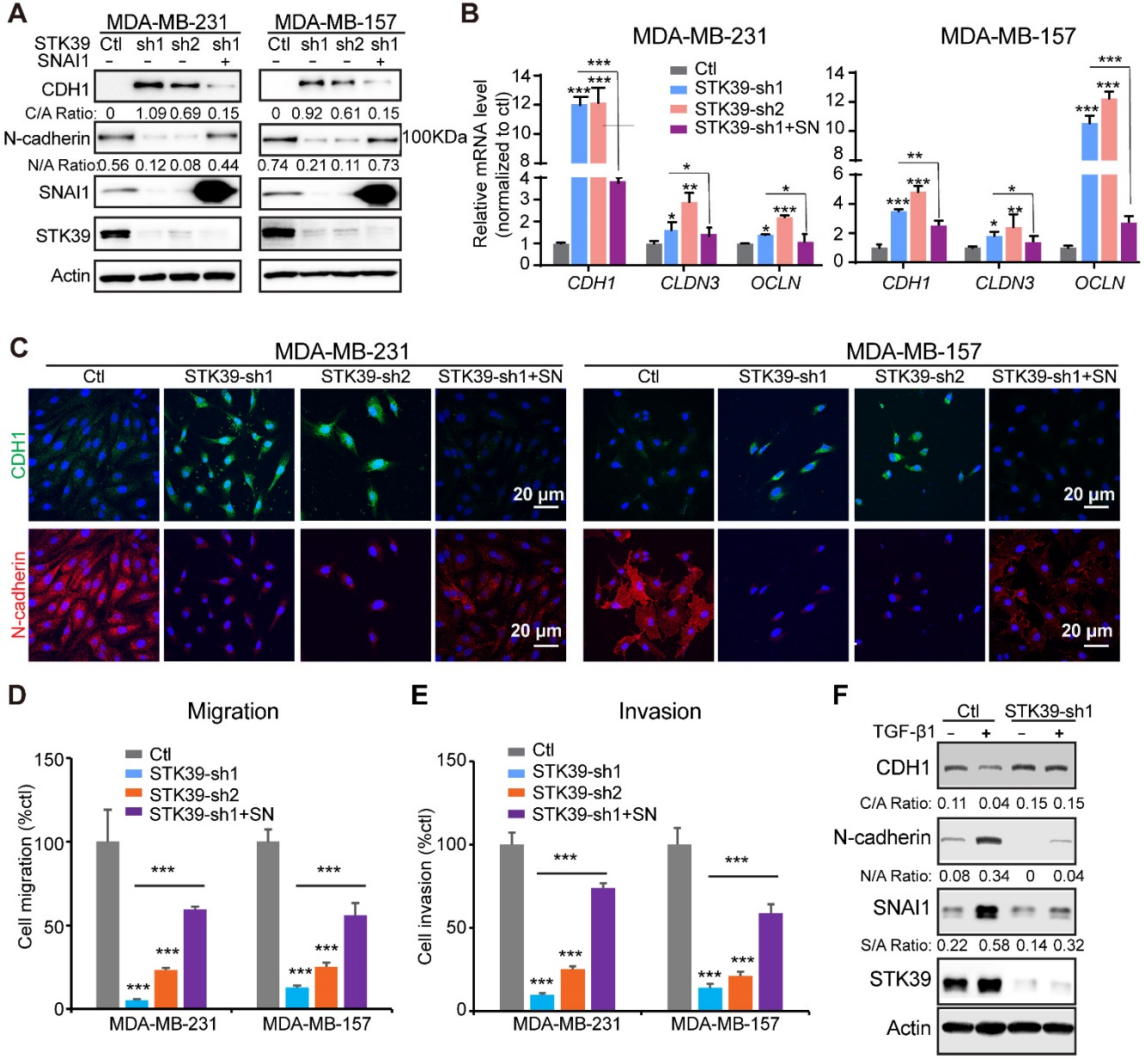
** Knockdown of STK39 inhibits EMT, migration and invasion in breast cancer cells. (A)** STK39 was knocked down by two different shRNAs in MDA-MB-231 and MDA-MB-157 cells. Rescued SNAI1 expression in the STK39-knockdown clone was also performed. The expression of CDH1, N-cadherin, STK39 and SNAI1 was analyzed by western blot. SN: SNAI1. **(B)** STK39 was knocked down by two different shRNAs in MDA-MB-231 and MDA-MB-157 cells. Rescued SNAI1 expression in the STK39-knockdown clone were also performed. The mRNA levels of epithelial markers were quantitated by real-time PCR. Data are the mean ± SD of two separate experiments in triplicates. CLDN3, Claudin 3; OCLN; Occludin. **(C)** Immnofluorescent images of EMT markers in MDA-MB-231 and MDA-MB-157 cells described in (**A**). CDH1 (green); N-cadherin (red); DAPI (blue). **(D)** Graphic representation of cell motility described in (**A**) analyzed by a migration assay. Data are the percentage of vector control values (mean ± SEM in three separate experiments in duplicates). **(E)** Graphic representation of cell invasion described in (**A**). Data are the percentage of vector control values (mean ± SEM in three separate experiments in duplicates). **(F)** MCF10A cells were transduced with control or shSTK39 lentiviruses. After selection in puromycin, cells were treated with TGF-β1 (2 ng/ml) for 2 days. The expression of CDH1, N-cadherin, STK39 and SNAI1 was analyzed by western blot. * P<0.05, **P<0.01, *** P<0.001.

To further assess the function of STK39 in breast cancer, we established clones with STK39 knockdown in MDA-MB-231 and MDA-MB-157 cells. We achieved 80-90% knockdown efficiency of endogenous STK39 using two independent shRNAs (**Figure 5A**). For both clones, STK39-knockdown increased CDH1 levels and downregulated expression of N-cadherin (**Figure 5A**). Consistent with this, loss of STK39 significantly increased mRNA expression levels of epithelial markers (**Figure 5B**). Immunofluorescence analysis also suggested an upregulation of CDH1 and downregulation of N-cadherin (**Figure 5C**). STK39 knockdown greatly inhibited the migration and invasive capabilities of these cells (**Figures 5D-5E, and S4**). Rescued SNAI1 expression in STK39-knockdown clones largely recovered the effects induced by STK39 ablation (**Figures 5 and S4**). The breast epithelial cells MCF10A were extensively used as a model to study the cellular EMT. As previously reported 19,37, TGF-β1 treatment induced EMT and activated expression of SNAI1 in MCF10A cells (**Figure 5F**). Depletion of STK39 significantly inhibited EMT and SNAI1 expression. Taken together, these results clearly suggest that STK39 enhances breast cancer metastasis, in large part, in SNAI1-dependent manner.

### STO phenocopies the effects of STK39 deficiency

**Figure 6:**
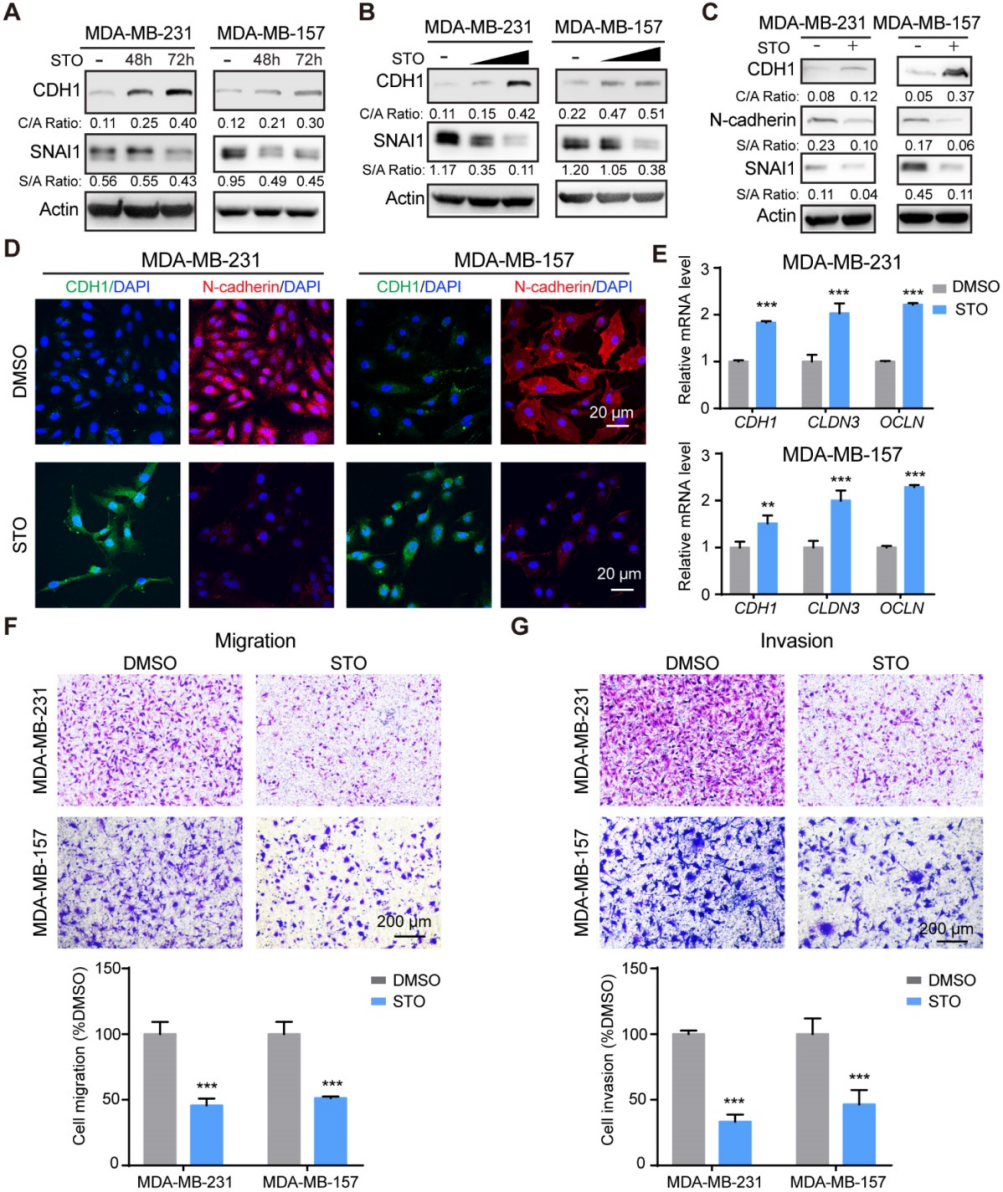
** STK39 inhibitor phenocopies STK39 deficiency. (A)** MDA-MB-231 and MDA-MB-157 cells were treated with STO 10 µM for different time intervals. Expression of endogenous CDH1 and SNAI1 were assessed by western blot. **(B)** MDA-MB-231 and MDA-MB-157 cells were treated with different doses of STO for 48 h. Expression of endogenous SNAI1 and STK39 were assessed by western blot. **(C)** MDA-MB-231 and MDA-MB-157 cells were treated with 10 µM STO for 48 h. The expression of CDH1, N-cadherin, and SNAI1 was analyzed by western blot. **(D)** Immunofluorescent images of EMT markers in MDA-MB-231 and MDA-MB-157 cell lines described in (**C**). **(E)** MDA-MB-231 and MDA-MB-157 cells were treated with 10 µM STO for 24 h. The mRNA levels of epithelial markers were quantitated by real-time PCR. Data are the mean ± SD of two separate experiments performed in triplicates. CLDN3, Claudin 3; OCLN; Occludin. **(F)** MDA-MB-231 and MDA-MB-157 cells were treated with 10 µM STO for 24 h and analyzed for cell migration. Representative imagines (upper panel) and graphic representation (lower panel) is the percentage of migration cells (mean ± SEM from three separate experiments in duplicates). **(G)** MDA-MB-231 and MDA-MB-157 cells were treated with 10 µM STO for 24 h and analyzed for cell invasion. Representative imagines (upper panel) and graphic representation (lower panel) is the percentage of invasive cells (mean ± SEM from three separate experiments in duplicate). STO, STOCK2S 26016 (STK39 inhibitor); ** P<0.01, *** P<0.001, compared with DMSO control group.

Treatment with STO increased CDH1 expression and downregulated SNAI1 expression in a time-course (**Figure 6A**) and dose-dependent manner (**Figure 6B**). STO treatment also up-regulated CDH1 levels and downregulated expression of N-cadherin (**Figure 6C**). Immunofluorescence analysis further revealed the increase of CDH1 and decrease of N-cadherin (**Figure 6D**). Consistent with STK39 deficiency, treatment with STO upregulated epithelial markers (**Figure 6E**) and greatly inhibited the migration and invasion of these cells (**Figures 6F-6G**). In sum, treatment with a STK39 inhibitor phenocopies the effects observed with loss of STK39 expression; specifically these include impaired migration, SNAI1 downregulation, and increased CDH1 expression, and thereby supports a critical role for STK39 kinase activity in EMT.

### Inhibition of STK39 sensitizes chemotherapy treatment and blocks metastasis *in vivo*

**Figure 7:**
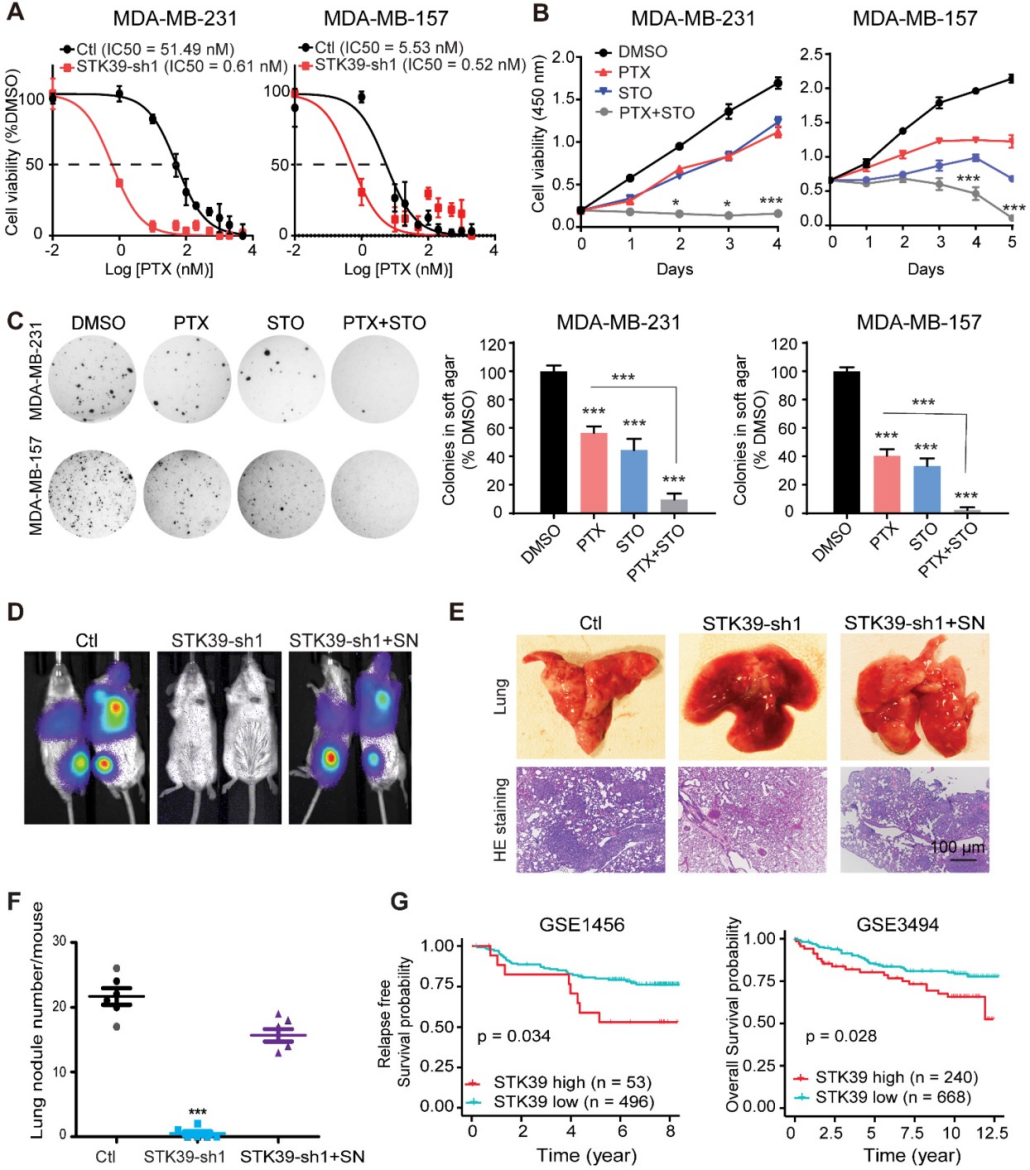
** Inhibition of STK39 sensitizes cancer cells to PTX treatment and suppresses tumor metastasis *in vivo*. (A)** MDA-MB-231 and MDA-MB-157 cells were transfected with control vector or STK39 shRNA to identify the IC50 of paclitaxel. **(B)** MDA-MB-231 and MDA-MB-157 cells were treated with 5 µM STO or 1µM PTX alone, or in combination for different time periods and cell proliferation was determined. **(C)** Cells were treated with DMSO, 5 µM STO or 1 µM PTX alone, or in combination for 24 h. Washed cells were plated in soft agar and cultured for 10-12 days. Colonies were fixed, stained and photographed. Presented data are the mean ± SD from three independent experiments. **(D)** MDA-MB-231-luc cells transfected with control, STK39 shRNA or STK39-knockdown cells with SNAI1 rescued expression were injected through tail vein into female SCID mice. Lung metastasis was assessed by bioluminescence imaging. Images are representative of each experimental group. SN, SNAI1. **(E)** Representative images of lung lesions (upper panel) and H&E stained lung sections (lower panel) from experimental groups in (**D**). **(F)** Lung nodule number from experimental groups in (**D**). **(G)** Kaplan-Meier plots of distant metastasis-free survival or overall survival of patients, stratified by expression of STK39. Data obtained from the GSE1456 and GSE3494 database. STO, STOCK2S 26016 (STK39 inhibitor); PTX, paclitaxel. * P<0.05, *** P<0.001, compared with control.

SNAI1 is associated with acquisition of chemoresistance 38. Paclitaxel (PTX) is the classical taxane, used in breast cancer therapy with efficacy in early and metastatic breast cancer 39. Unfortunately, effective and successful therapy for patients is commonly limited by an acquired resistance. To test whether knockdown of STK39 enhances paclitaxel sensitivity, we determined the IC50 of PTX with or without depletion of STK39 in MDA-MB-231 and MDA-MB-157 cell lines. Loss of STK39 reduced the IC50 of PTX (**Figure 7A**). To determine whether STO has synergy with PTX, we treat the MDA-MB-231 and MDA-MB-157 cells with paclitaxel with or without STO treatment. STO acted synergistically with paclitaxel to suppress cell proliferation (**Figure 7B**). Soft agar colony formation analysis revealed that the combination treatment with STO and PTX resulted in a greater reduction in both colony formation and colony size when compared to paclitaxel alone (**Figure 7C**). Our data suggest that STK39 inhibition sensitizes breast cancer cells to PTX.

To directly assess whether STK39 is critical for cell metastasis *in vivo*, we intravenously injected STK39-knockdown MDA-MB-231-luciferase cells into female SCID mice and subjected these mice to bioluminescent imaging (BLI). All control mice were moribund due to massive lung metastases (**Figure 7D**). In contrast, mice injected with STK39-knockdown cells were viable and free of detectable metastases. Control cells exhibited a high number of metastatic lesions whereas STK39-knockdown cells lacked metastatic colonies by histologic analyses (**Figures 7E-7F**). In agreement with the function of SNAI1 *in vitro*, expression of exogenous SNAI1 in STK39-knockdown cells largely rescued the formation of lung metastasis (**Figures 7D-7F**). SNAI1 expression was inversely correlated with tumor-free survival in breast cancer 40. To verify whether women with primary breast cancers that express a high level of STK39 relapse at a faster rate than women whose breast cancers express a low level of STK39, in a pattern similar to that of SNAI1, we analyzed two microarray expression datasets derived from primary human breast cancers in which both STK39 expression level and clinical outcome were available. Intriguingly, individuals with high STK39 expression had a reduced overall survival or interval of disease-free survival (**Figure 7G**). These results suggest that STK39 expression may represent an important prognostic indicator for breast cancer in the clinical setting.

## Discussion

Our current study demonstrates that STK39 stabilizes SNAI1 through phosphorylation at T203, which is critical for its nuclear retention. In addition, STK39 plays a critical role in tumor metastasis. More importantly, STK39 knockdown cells are more sensitive to chemotherapeutic treatment. Also, STK39 expression correlates with poor survival of breast cancer patients. Therefore, our study not only uncovers a role for STK39 in breast cancer progression but also provides new insights into the regulation of SNAI1.

SNAI1 stability is extensively regulated by phosphorylation. On one hand, phosphorylation of SNAI1 promotes its proteasomal-mediated ubiquitination degradation. For example, both CK1 and DYRK2-mediated SNAI1 phosphorylation act as a prime phosphorylation that permits GSK3β-mediated phosphorylation, leading to β-TRCP-induced poly-ubiquitination and degradation 12,41. PKD1-mediated phosphorylation of SNAI1 facilitates FBXO11-mediated SNAI1 degradation 42. Under intact apical-basal polarity, aPKC kinases promote degradation through phosphorylation of SNAI1 S249 43. Other phosphorylations of SNAI1 prevent its degradation. Most commonly, SNAI1 ubiquitination is blocked by reducing its affinity for GSK3β. For example, phosphorylation of SNAI1 by ATM and DNA-PKCs inhibits SNAI1 ubiquitination through reducing its interaction with GSK3β 20,22. Recently, it was shown that p38 stabilizes SNAI1 through phosphorylation at Ser107, which suppresses DYRK2-mediaed Ser104 phosphorylation that is required for the GSK3β-mediated SNAI1 degradation 18. Alternatively, SNAI1's confinement in the nucleus prevents degradation. Both PI3K and PAK1 phosphorylate SNAI1 on Ser246 to increase SNAI1's accumulation in the nucleus 44,45. ERK2-mediated Ser82/Ser104 phosphorylation of SNAI1 leads to SNAI1 nuclear accumulation 21. Lats2 phosphorylates SNAI1 at T203 in the nucleus, which prevents nuclear export, thereby supporting stabilization 19. In this study, we found that STK39 also enhances SNAI1 stability by its phosphorylation at T203. Notably, Lats2 directs its association at the N-terminal region (aa 10-40) of SNAI1 19 whereas STK39 interacts with C-terminal region which harbors T203, raising the potential that Lats2 and STK39 can simultaneously interact with and phosphorylate SNAI1 at T203. STK39 promotes SNAI1 stability by blocking protein degradation but does not decrease its poly-ubiquitination (data not shown). We also noticed that STK39 did not impair the interaction between GSK3β and SNAI1. A plausible explanation for this confounding observation could be that STK39 phosphorylates SNAI1 at T203 promoting accumulation in the nucleus, whereas the GSK3β-degradation components are cytoplasmic. However, the precise molecular mechanisms for the T203 phosphorylation-mediated SNAI1 stability remain to be fully elucidated.

Multiple studies showed that STK39 plays key roles in regulating cellular ion homeostasis and blood pressure through activation of NCC and NKCC2 30. STK39 also increases colonic epithelial permeability and pro-inflammatory cytokines, and STK39 knockout mice lack intestinal and renal inflammation and pro-inflammatory cytokine secretion compared to control mice 27,46. However, recent studies identify functions in cancer progression as well. Reports demonstrated that STK39 expression was significantly increased in non-small lung cancer cells, and expression was positively associated with advanced tumor staging, lymph node metastasis and poor prognosis 29. However, the underlying mechanism was unknown. It was also reported that STK39 was necessary for proliferation but not for endothelial cells migration 47. Our studies clearly showed that STK39 controls EMT by stabilizing the CDH1 repressors of SNAI1 and is crucial for migration in breast cancer. Accordingly, loss of STK39 expression inhibited the migration and invasion of cells *in vitro* and metastasis *in vivo*. Therefore, our study not only clearly confirms the role of STK39 in tumor metastasis but also reveals the underlying molecular mechanism. Notably, STK39 protein levels did not correlate with the SNAI1 protein expression in breast cancer cell lines and breast cancer tissues (data not shown) because the activity of STK39 need to be activated. However, no suitable marker is available to detect the STK39 activation.

Because of its unique structural organization and important roles in regulating blood pressure, kidney disease, and cancer, STK39 is an active drug target that may hold future promise. Indeed, compounds that inhibit STK39 activity have been developed and show promise as a potential anti-cancer drugs 48-50. STOCK2S 26016 (STO), a novel compound developed by high-throughput screening, inhibits STK39 activation by reproducibly disrupting the binding of WNK to STK39 51. We found that STO phenocopied the effect of STK39 deficiency and inhibited cancer cell migration and invasion *in vitro*. In addition, the combined administration of STOCK and paclitaxel produced a synergistic therapeutic effect. However, there are no clinically-approved drugs that target STK39 being used to treat cancer. The pharmacokinetics and pharmacodynamics of STOCK are unknown 24. Additional investigations are required to initiate live animal experiments, and then examine use clinically. Such action would offer synergistic effects with chemotherapy treatment on human breast cancer treatment.

In summary, our study unveils a mechanism by which STK39 promotes EMT and the metastasis of tumor cells by enhancing the stability of SNAI1. Our study extended the multifaceted role STK39 in human disease from that of a key regulator of hypertension to a key metastasis promoter. Our study also has important implication for the development of STK39-based targeting strategies for metastatic cancers.

## Supplementary Material

Supplementary figures.
