# Supplementary material for: STK39 promotes breast cancer invasion and metastasis by increasing SNAI1 activity upon phosphorylation
Source: Theranostics. 2021 Jun 11;11(16):7658–70. doi: 10.7150/thno.62406 (PMC8315073; doi:10.7150/thno.62406)
Supplement: Supplementary file 1 — Supplementary figures. [file thnov11p7658s1.pdf]

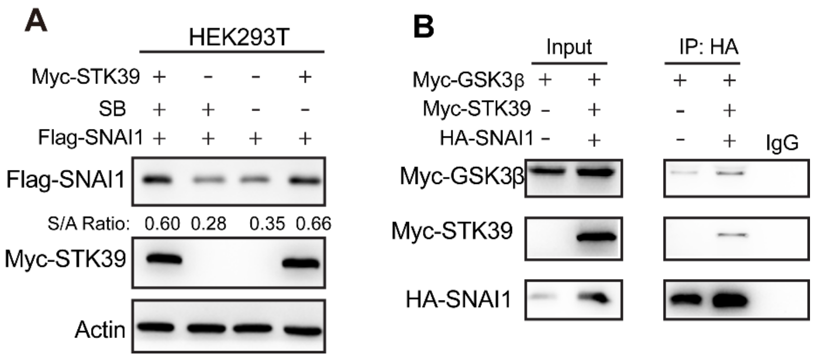

Supplemental Figure 1: (**A)** Flag-Snail was expressed with or without Myc-SPAK. Cells were treated with or without 10 μm SB203580 (SB) for 24 h. Lysates were analyzed by western blot. **(B)** HA-Snail and Myc-GSK3β was co-transfected with or without Myc-SPAK into 293T cells, then treated with MG132 for 6 h. Cell lysates were immunoprecipitated using anti-Myc antibody and analyzed by immunoblotting.

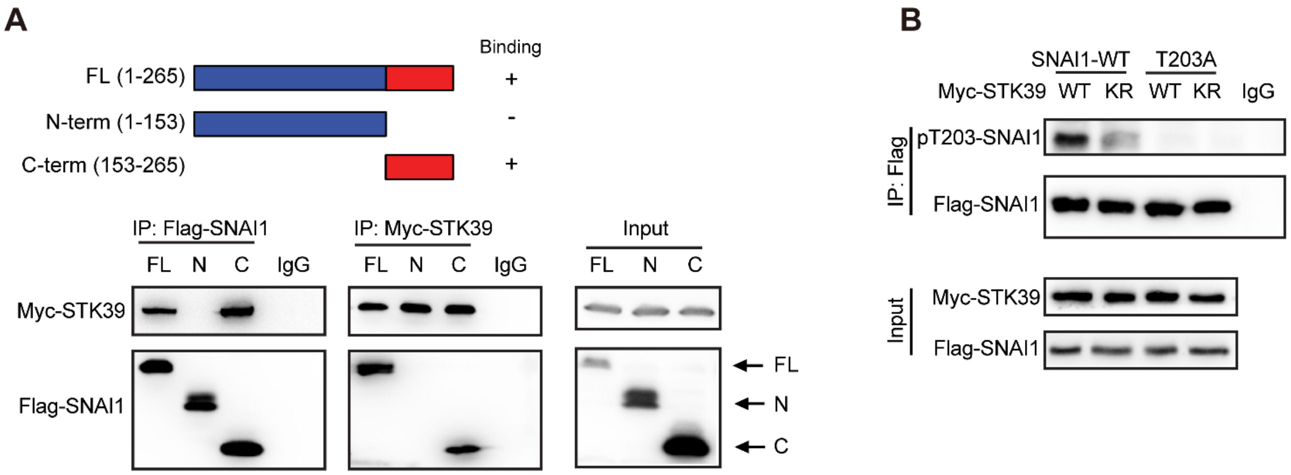

Supplemental Figure 2: (A) Schematic diagram showing the structure of Snail and deletion constructs used (top panel). Flag-tagged full-length (FL) or deletion mutants of Snail were co-expressed with Myc-SPAK in HEK293T cells. Extracts were subjected to IP with Flag or Myc antibody, and bound SPAK or Snail was analyzed by Western blot using either Myc or Flag antibody. **(B)** Flag-Snail WT or Flag-Snail T203A was co-transfected with Myc-SPAK WT or T203 into 293T cells, then treated with MG132 for 6 h. Cell lysates were immunoprecipitated using anti-Flag antibody and analyzed by immunoblotting using a specific antibody against pT203-Snail.

A

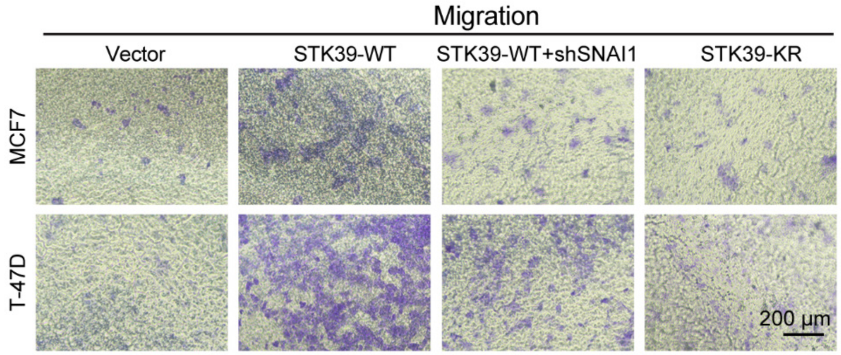

B

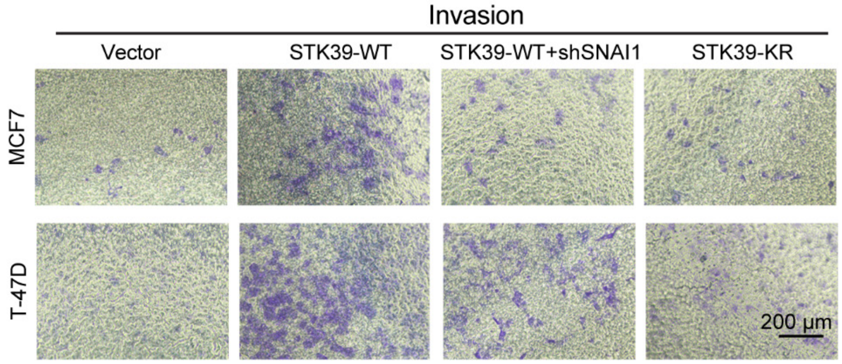

Supplemental Figure 3: (**A)** STK39 was expressed in MCF7 and T-47D cells. A rescue experiment with knockdown of SNAI1 expression in WT-STK39 expressing cells was also performed. Representative imagines for cell migration. **(B)** Representative imagines for cell invasion.

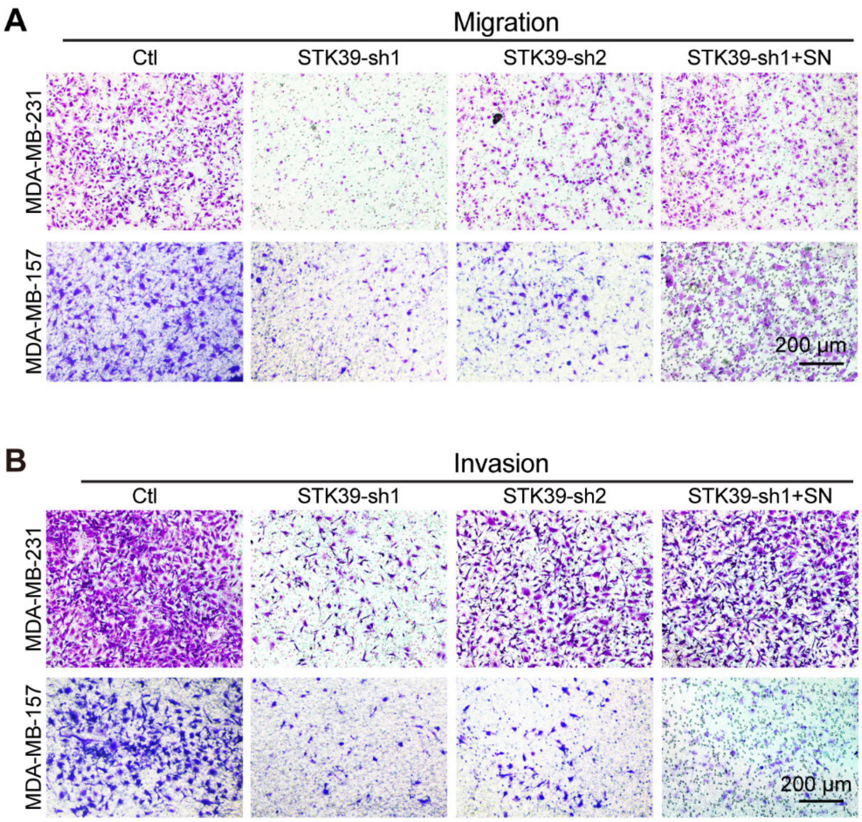

Supplemental Figure 4: **(A)** STK39 was knocked down by two different shRNAs in MDA-MB-231 and MDA-MB-157 cells. Rescued SNAI1 expression in the STK39-knockdown clone was also performed. Representative imagines for cell migration. **(B)** Representative imagines for cell invasion.
